# Blue tits are outperformed by great tits in a test of motor inhibition, and experience does not improve their performance

**DOI:** 10.1098/rsos.221176

**Published:** 2023-02-22

**Authors:** Utku Urhan, Magnus Mårdberg, Emil Isaksson, Kees van Oers, Anders Brodin

**Affiliations:** ^1^ Department of Biology, Lund University, Lund, Sweden; ^2^ Netherlands Institute of Ecology (NIOO-KNAW), Wageningen, The Netherlands; ^3^ Department of Biology, University of Ottawa, Ottawa, Canada

**Keywords:** motor inhibition, transparent cylinder task, Paridae, transparency experience

## Abstract

Motor inhibition refers to the ability to inhibit immediate responses in favour of adaptive actions that are mediated by executive functions. This ability may be an indication of general cognitive ability in animals and is important for advanced cognitive functions. In this study, our aim was to compare motor inhibition ability of two closely related passerines that share the same habitat. To do this, we tested motor inhibition ability using a transparent cylinder task in blue tits in the same way as we previously tested great tits. To test whether the experience of transparent objects would affect the performance of these species differently, both in the present experiment using blue tits and our previous one on great tits, we divided 33 wild-caught individuals into three different treatment groups with 11 birds each. Before the test we allowed one group to experience a transparent cylindrical object, one group to experience a transparent wall and a third group was kept naive. In general, blue tits performed worse than great tits, and unlike the great tits, they did not improve their performance after experience with a transparent cylinder-like object. The performance difference may stem from difference in foraging behaviour between these species.

## 1. Introduction

In animals, motor inhibition refers to the ability to inhibit immediate behavioural responses in favour of more adaptive actions, mediated by so-called executive functions [1,2]. Motor inhibition belongs to a group of top-down mechanisms known as executive functions that are essential for many complex cognitive processes, such as problem solving, decision making and planning [3,4]. Executive functions are considered to be a domain-general cognitive ability that are involved in tasks in other cognitive domains, unlike domain-specific abilities, for which the functions have evolved for a specific purpose [5]. Several comparative studies have shown that species vary in their mean level of motor inhibition [4,6–8] and comparisons of this ability between different species can yield important insights into the evolution of complex cognition. To make such comparisons possible it is essential to test different species in a consistent, standardized way.

A common way to test inhibitory motor control is using a transparent cylinder task. In this task, the subjects are exposed to a food reward in a transparent cylinder that is positioned perpendicularly to the direction of the animal's approach. If the subject tries to reach for the reward directly through the transparent wall of the cylinder, it is considered a failure. If it instead inhibits this non-adaptive behaviour and takes the reward by going to the open end of the cylinder without touching the wall, the subject is considered to have passed the test. A subject that passes the test is hence considered to possess a level of motor self-regulation that allows inhibition of movement [2,9].

The number of species that have been tested in the transparent cylinder task is growing and it is generally believed that performance in this task is positively correlated with the general cognitive ability [4,6]. However, studies do not always yield straightforward patterns. For example, guppies *Poecila reticulata*, which are generally assumed to have lower general cognitive ability due to their relatively small brain size compared with passerine birds and mammals, have been reported to perform on a similar level as many small passerines and mammals when tested in a transparent barrier task that works for fish and is considered to correspond to the transparent cylinder task [7]. Also, four species of large parrots that are known for their high cognitive abilities [10–12] performed worse than some small passerines [6,8] and guppies [7]. To what degree the properties of the transparent object and the familiarity with the transparent object that is used for the task influences the performance remains unclear. For example, previous experience of transparent objects and motivation played a role in determining individual performance in pheasants [13]. In line with this, In a previous experiment, we showed that great tits that have previous experience of a transparent cylinder performed better than control birds or birds that only had experience of a transparent wall [8].

Not only great tits but also blue tits are arboreal habitat generalists that have successfully colonized urban environments. This is important, as inhabiting urban environments is likely to require a high level of behavioural flexibility, which has been suggested to correlate with cognitive ability [14]. Both anecdotal and empirical evidence suggests that both blue and great tits are innovative foragers. For example, both species were known to pierce milk bottle caps outside people's homes, in order to reach the cream on the top [15]. In some studies, this behaviour is primarily reported for blue tits even though the original inventor of this behaviour has always been great tits when this has been possible to verify [16]. Great tits have been shown to perform various tasks that are considered to require advanced cognitive skills [17–19]. There are few studies that compare these two species in the same set-up. In one of those, great tits outperformed blue tits in an observational learning task which tests the social cognition performance in animals [20]. However, to our knowledge, the performance of these two species has not been compared in a task testing domain general cognitive ability, such as motor inhibition. Such comparisons of closely related species can add to discussion of evolution of cognitive skills and adaptations.

In this study, we tested blue tits (*Cyanistes caeruleus*) in the cylinder task in the same way as our group has previously tested its close relative, the great tit (*Parus major*) [8]. Just like in our previous study, we divided the birds into three treatment groups, differing in their previous experience of transparency, to investigate whether previous knowledge about transparency had any effect on performance. If there is a difference in the ability of motor inhibition between these two species our prediction is that great tits will perform better than blue tits in accordance with the performance of these species when it comes to observational learning ability [20].

## 2. Methods

As the main purpose of this study was to compare performance in blue and great tits, we merged our blue tit data with the great tit dataset from our previous study [8]. To make comparisons as fair as possible, we replicated the methods from that study as closely as possible. We tested the same number of birds under as identical conditions as possible in the same facility. Conditions in the facility were the same with respect to temperature, light regime, food provided, positions of the bird's home cages and experimental apparatus and devices (see [8] for details).

### 2.1. Subjects

We captured 33 blue tits (17 male, 16 female) between early September 2020 and February 2021, around Höör and Lund in southern Sweden, using mist nets and playback of great tit and mixed flock songs. The sites around Höör are situated in deciduous forests, whereas the ones around Lund are suburban, such as city parks and residential gardens. As we did not find any habitat effect in our great tit data, we did not consider habitat in this study. We captured the blue tits at the same locations as we previously had captured the same number of great tits. All individuals were adults, that is, in their second calendar year or older. Transportation time from the capture site to our indoor animal facility never exceeded 30 min. During transportation, we kept the birds in individual cotton bags of the type that is normally used for bird ringing. Birds were in captivity under permit M-213-11 from the Malmö-Lund regional ethical permit board.

After bringing the birds to the laboratory, we immediately transferred them to individual cages, made of metal bars, measuring 60 × 60 × 40 cm. We kept the birds in these cages during the rest of their stay in the laboratory, except for during the experimental sessions. The cages were placed on shelves in the experimental room. Since blue tits are a flock-dwelling species, we placed two cages on each shelf so that each bird could have close visual and vocal contact with another individual from the same winter flock.

The birds had ad libitum access to water and food in their home cage. The main food source consisted of a mixture of sunflower seeds, peanuts and hempseeds. We also provided lard cakes as additional food. A few birds did not start eating directly in the laboratory. We provided these with living mealworms which usually works as a ‘jump-starter’ that will make unwilling birds motivated to start eating. With this procedure all birds ate readily for their whole stay in the laboratory. After completion of the experiments, we released the birds at the same location where they had been captured. Before we released the birds, we made sure that they were in good condition.

### 2.2. The experimental facility

The experimental room was 5 m long, 3 m high and 2.6 m wide, located in an animal facility at the Department of Biology at Lund University. The temperature and the light regime in these rooms were computer controlled; the temperature being kept constant at 14°C and the day length changing in approximate accordance with the outdoor conditions (it varied between 10/14 h and 8/16 h light/dark). The lights in the laboratory had a daylight spectrum and a 1-hour dimming function that made it possible to simulate dawn and dusk.

Before each experimental session, we moved the birds from their home cage to a special experimental cage (see below). During experimental sessions, the observer sat in an observation booth that was covered by smoke-coloured glass that allowed free view of the focal bird without being seen.

We used the same experimental cage as we previously used in the great tits study [8]. It was of the same type as the birds' home cages (60 × 60 × 40 cm) but equipped with an attached experimental box, measuring 36 × 21 × 25 cm. The box was made of wooden boards on the sides with an open side towards the bird's cage and a transparent Plexiglas wall on the side facing the experimenter. A slide door that could be opened or closed by pulling a string allowed passage for the bird between the cage and the box. This made it possible to control the bird's access to the experimental box without handling. At the bottom of the box there was a small rotatable platform that the experimenter could turn horizontally in either direction by pulling one of two strings (see below). Hence the birds had full view of the inside the box from the cage. We recorded all training and experimental sessions with a Sony Action Cam HDR-AS200VT.

### 2.3. Experimental procedure

We allocated the birds to one of three treatment groups. For group one (*n* = 11, 5 females and 6 males), we mounted a transparent plexiglass wall measuring 17 × 17 cm in the home cage for 3 days before the experiment. These birds thus had prior experience of transparency in general but not specific experience of a transparent cylinder. For group two (*n* = 11, 4 females and 7 males) we placed a transparent plexiglass cylinder at the bottom of their home cage for 3 days before the experiment. This cylinder was similar to the experimental one, which measured 9 × 4 cm with an inner diameter of 3.3 cm, but was slightly smaller (9 × 3 cm, inner diameter 2.3 cm) and it contained no food. Hence, these birds had experience with a transparent cylinder. Group three (*n* = 11, 7 females and 4 males) was control birds with no experience of any type of transparency as far as we know. Henceforth we refer to these birds as naive.

For the training session and the test sessions, birds were moved to an experimental cage where they were provided with water but no food except for the reward that they could obtain in the task. In the training session, the birds were allowed to retrieve a mealworm that was hidden in a white opaque plastic cylindrical tube with its openings placed perpendicular to the direction of the birds' approach. The cylinder had two openings which made the reward accessible from both ends of it, but not from the direction from which the bird approached. We started the first training session by placing the opaque cylinder on the rotatable platform, in the centre of the box. When perching in the cage the birds could not see the content inside the cylinder, but they could see that there were openings on both sides of it. Before we allowed the bird to enter the wooden box, we rotated the platform and the cylinder to show the bird the mealworm. After 30 s, we rotated the cylinder back to its original position. Then we allowed the bird access to the box to retrieve the reward. If a bird managed to retrieve the reward within 6 min, we considered it a successful trial, although we allowed the bird to retrieve the reward regardless of the time. We performed a maximum of five trials a day as some of the birds appeared to lose motivation after that. The rotation procedure was only done in the first training session and was not repeated in consecutive training or test sessions. When a bird succeeded to retrieve the mealworm from the cylinder within 6 min in four consecutive sessions, we allowed the bird to proceed to the test sessions. All 33 birds that were involved in our study passed this criterion.

We started the test sessions one day after a bird had successfully completed its training sessions. We conducted a total of 10 trials over two consecutive days, with each individual being tested in five trials, with 20 min breaks in between, in one day. The rationale for dividing 10 trials into two consecutive days was to keep birds motivated to participate in the task. In the test sessions, we used a transparent cylinder instead of an opaque one. As in the training sessions, we had placed a reward in the centre of the cylinder, which in turn, was placed in the wooden box. This means that the birds had a full view of the cylinder and the reward from the main cage. In an experimental session, we allowed the focal bird to enter the wooden box from the main cage and recorded its behaviour. If the bird pecked at the wall of the transparent cylinder before retrieving the reward, we considered this a failed attempt. On the other hand, if it first moved to an opening of the cylinder and retrieved the reward from there without touching the cylinder, we considered this to be a successful attempt. The performance was calculated as the proportion of successful attempts out of 10 sessions.

### 2.4. Statistical analyses

We merged our data on 33 blue tits with the data of the 33 great tits that was previously collected. We used the R statistical software [21] for statistical testing of our results. To test if species and experience influenced motor inhibition, we used a binomial generalized linear model (GLM) (g*lm* function in R) [22] with the *cbind*() function including the total number of successful attempts in relation to total number of trials as the response variable, and species (blue tit and great tit) and experience (naive, cylinder and wall) and their interaction as fixed factors. In order to specifically test for an effect of experiencing a transparent cylinder, we ran a *post hoc* analysis with Bonferroni correction on the interaction between species and experience. We were specifically interested in the contrasts between cylinder-experienced blue tits and great tits, and naive blue tits and great tits. Using a LM with the number of successful trials within 10 trials as the response variable, provided very similar results (see the R code and the data in the electronic supplementary material). In order to conduct a *post hoc* analysis of our models, we used the *emmeans* function in the emmeans package in R [23]. In order to investigate whether birds’ performance improved over trials and whether this improvement depended on species and treatment group, we performed another GLM, where we used success of each individual in each trial as a response variable, and trial number, species, type of experience prior to testing and their interaction as fixed factors.

## 3. Results

**Figure 1.**
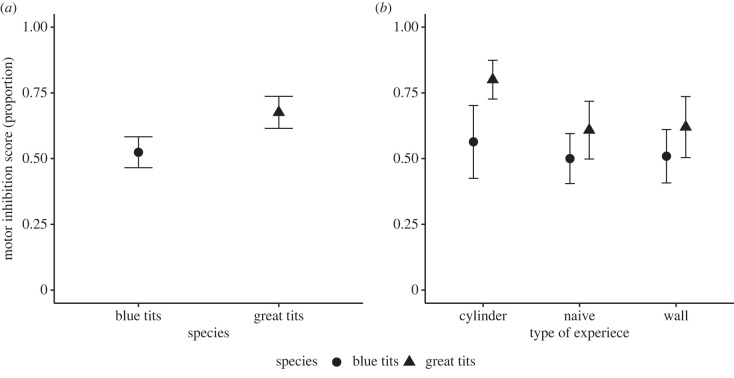
(*a*) The average proportion of correct attempts in the transparent cylinder task for each species, and (*b*) the average proportion of correct attempts separated by treatment groups. Filled circles represent blue tits and filled triangles represent great tits. The error bars are 95% confidence intervals.

**Figure 2.**
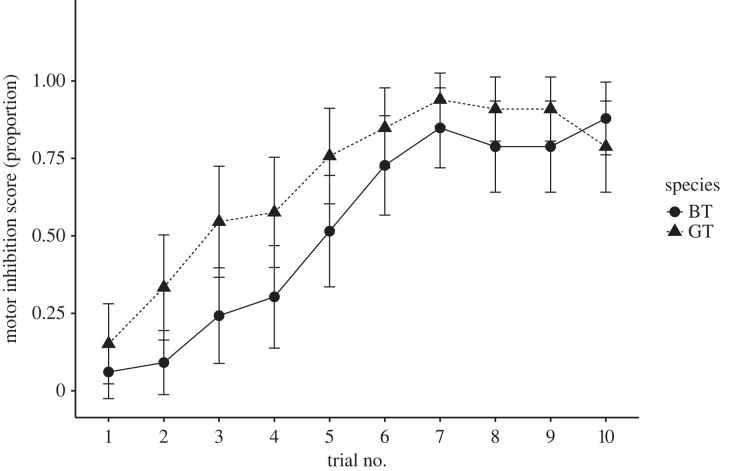
The average proportion of correct attempts for all birds in each session. Filled circles with straight lines represent blue tits and filled triangles with dashed lines represent great tits. The error bars are 95% confidence intervals.

When comparing all individuals, great tits performed better (0.68 ± 0.17) than blue tits (0.52 ± 0.17) in the proportion of correct attempts on the cylinder task, which is a classic measure of motor inhibition (figure 1*a*, $\chi_{2\;}^{2}=15.86$, *p* < 0.001). Type of experience (treatment groups; naive, cylinder or wall) had a significant effect ($\chi_{2\;}^{2}=9.6505$, *p* = 0.008) and the interaction between species and treatment group was not significant ($\chi_{2\;}^{2}=3.64$, *p* = 0.16). However, since we were specifically interested in the contrast between blue tits and great tits that had experience with the transparent cylinder, we conducted a *post hoc* test, which showed that cylinder-experienced great tits performed significantly better (tables 1 and 2, *p* = 0.003) compared with cylinder-experienced blue tits (figure 1*b*), while there was no difference in the performance between the three treatment groups in blue tits (figure 1*b*, table 2), indicating that experience with a transparent cylinder did not improve performance in blue tits. There was a general increase in bird's performance over trials (figure 2, $\chi_{9\;}^{2}=229.84$, *p* < 0.001) but the three-way interaction between trial number, type of experience and species ($\chi_{18\;}^{2}=23.92$, *p* = 0.16) was not significant, suggesting that great tits and blue tits from different treatment groups improved their performance similarly (table 3).

**Table 1.** Relation between species, type of transparent object experience prior to trials and transparent cylinder task success.

factors	delta deviance	d.f.	*p* value
species	15.86	1	0.000068***
type of experience	9.65	2	0.008**
species * type of experience	3.64	2	0.16

**Table 2.** The *post hoc* test that compares performance in the transparent cylinder test in relation to species and treatment group. Statistical values come from the summary () function. ‘naive’ indicates no experience of any kind prior to testing, ‘cylinder’ indicates transparent cylinder experience prior to testing, ‘wall’ indicates transparent wall experience prior to testing, ‘BT’ indicates blue tits and ‘GT’ indicates great tits. *p* values are adjusted using ‘Bonferroni method’.

comparison	odds ratio	s.e.	*z* ratio	*p* value
BT cylinder–GT cylinder	0.323	0.0989	−3.691	0.0033**
BT cylinder–BT naive	1.292	0.3498	0.945	1.0000
BT cylinder–GT naive	0.832	0.2231	−0.687	1.0000
BT cylinder–BT wall	1.246	0.3373	0.811	1.0000
BT cylinder–GT wall	0.792	0.2231	−0.829	1.0000
GT cylinder–BT naive	4.000	1.2210	4.541	0.0001***
GT cylinder–GT naive	2.575	0.7803	3.122	0.0269*
GT cylinder–BT wall	3.857	1.1775	4.422	0.0001***
GT cylinder–GT wall	2.452	0.7724	2.846	0.0664
BT naive–GT naive	0.644	0.1720	−1.649	1.0000
BT naive–BT wall	0.964	0.2601	−0.135	1.0000
BT naive–GT wall	0.613	0.1721	−1.744	1.0000
GT naive–BT wall	1.498	0.4001	1.512	1.0000
GT naive–GT wall	0.952	0.2649	−0.177	1.0000
BT wall–GT wall	0.636	0.1784	−1.614	1.0000

**Table 3.** Generalized linear model that tests the relation between success in each session for each species and transparent object experience prior to trials.

factors	delta deviance	d.f.	*p* value
type of experience	9.36	2	0.0093**
trial	229.84	9	< 0.001 ***
species	24.21	1	<0.001 ***
type of experience * trial	26.11	18	0.097
type of experience * species	3.27	2	0.20
trial * species	7.78	9	0.56
type of experience * trial : species	23.92	18	0.16

## 4. Discussion

Great tits performed better than blue tits on the cylinder task, which is in line with previous studies that have shown great tits perform better than blue tits in other cognitive tasks, both under laboratory conditions [20] and in the wild [24]. Furthermore, when both species were exposed to the same cylindrical transparent object prior to the task, great tits improved their performance, while this was not the case for blue tits. This suggests that cognitive abilities may differ even between closely related species that live in the same environment, frequently occur in mixed species groups and experience similar selection pressures.

Even though blue tits performed worse that great tits (52% and 68%, respectively), it should be noted that they outperform some other bird species that have been tested in the same task, such as the song sparrow (*Melospiza melodia*) 26.5%, the swamp sparrow (*Melospiza georgiana*) 26.1%, the African grey parrot (*Psittacus erithacus*) 34%, the blue-headed macaw (*Primolius couloni*) 33% and the domestic chicken (*Gallus gallus*) 31.33% [6,25,26]. Both great tits and blue tits are known to be highly flexible, quick at adapting to both new natural and anthropogenic habitats, as both species are generalist foragers. Therefore, both species may frequently have to adopt new foraging skills, requiring good understanding of the environments in which they live.

Why then should there be a difference in cognitive performance between great and blue tits? The variation in adaptations can occur even though animals are closely related and ecologically similar [27]. Although they are closely related and live in the same environment, blue tits and great tits exhibit differences in their behaviour. For example, great tits will more frequently investigate feeders from a distance before flying to them, whereas blue tits tend to fly directly to them [28]. Inhibiting the urge to fly towards the food source and wait a few seconds before approaching could allow an animal extra time to scan for predators and to make a better decision on how to forage efficiently. The two species also differ in their foraging styles. For example, it has been shown that great tits benefit from being in a mixed-species flock in terms of foraging efficiency [29] and can exploit wider selection of food sources compared with blue tits [30]. These differences in foraging styles may require great tits to have higher level of general cognitive ability compared with blue tits.

Perhaps the most interesting result of our study is that great tits improved their performance after having experience with a transparent cylinder whereas blue tits showed no improvement. Although this should be interpreted with caution, it agrees with a study by Sasvári [20], in which it was demonstrated that great tits are better learners than both blue tits and marsh tits *Poecile palustris* in an observational learning task, where the birds had to use the information they had acquired by observation prior to the test [20]. It has also been shown that these two species differ in their level of innate versus learned behaviours. In a comparative study, naive hand-raised great tits show no sign of neophobic behaviour toward aposematic stimuli and hence had to learn to avoid aposematic prey. Hand-raised blue tits, on the other hand, demonstrated an innate avoidance behaviour toward aposematic stimuli [31]. Hence behaviour that is innate in blue tits had to be learned by great tits. In general, learned behaviours will be more flexible than innate ones, but learning will also require more advance cognitive abilities [32].

Although a potential year-effect may confound some of the species differences, since blue tits and great tits were tested in different years, the risk that our results are caused by a difference in the harshness of the environment between years is very small. Birds were acclimatized in captivity for at least 5 days, which diminished an immediate possible hunger effect. Moreover, birds within species varied in age, which probably evened out experienced variation in harshness over years. Another alternative explanation to our results could be that blue tits did not interact with the transparent objects in their cage, but great tits did. We could not test for this; however, parids are curious birds and typically when kept in captivity they investigate the objects around them. The transparent objects stayed in the cages for several days for both species, which increased the likelihood of interaction.

In conclusion, blue tits performed worse than great tits in the cylinder task that tests for motor inhibition but both performed better compared with other small passerine species [6]. Motor inhibition ability has been suggested to be an important top-down mechanism that is involved in several complex cognitive mechanisms. Furthermore, only great tits improved performance by learning, which supports previous studies that suggest that great tits are better learners than blue tits.

## Data Availability

The data presented in the study are made available in the article's supplementary materials section [33]. Further enquiries concerning data and the codes can be directed to the corresponding author.
